# CC-115 Mediates GSDME-Dependent Pyroptosis in Lung Adenocarcinoma Through the Akt/Bax Pathway

**DOI:** 10.7150/jca.83175

**Published:** 2023-05-15

**Authors:** Ting Zhang, Ming-Quan Liu, Guang-Su Xie, Dong-Ming Wu, Peng-Wei Luo, Teng Liu, Shi-Hua Deng, Yuan-Yi Wang, Shuang He, Ye Zhou, Jin Zhou, Ying Xu

**Affiliations:** 1 School of Clinical Medicine & The First Affiliated Hospital of Chengdu Medical College, Chengdu 610500, China.; 2 Xindu District People's Hospital of Chengdu, Chengdu 610500, China.; 3 Sichuan Cancer Hospital and Institute, School of Medicine, University of Electronic Science and Technology, Sichuan 610042, China.

**Keywords:** Bax, pyroptosis, CC-115, GSDME, lung adenocarcinoma, chemotherapy

## Abstract

Chemotherapeutic agents remain the first-line treatment for solid tumors, including lung cancer, but chemotherapy resistance is hampering global efforts to treat this disease. CC-115 is a novel antitumoral compound used in phase I clinical trials. However, it is unclear whether CC-115 is effective against lung adenocarcinoma (LUAD). In the present study, we found that CC-115 induced lytic cell death in A549 and H1650 tumor cells via swelling of cells and formation of large bubbles on the plasma membrane that closely resembled those typical of pyroptosis, a type of programmed cell death linked to chemotherapy. We demonstrated that CC-115 exerts antitumor effects in LUAD through gasdermin E (GSDME)-mediated pyroptosis by acting as a dual inhibitor of DNA-PK and mTOR. CC-115 can inhibit Akt phosphorylation, impairing its inhibitory effect on Bax, thereby inducing pyroptosis via the Bax-mitochondrial intrinsic pathway. CC-115-induced pyroptosis was abrogated by treatment with the Akt activator SC79 or by depletion of Bax. Importantly, CC-115 significantly upregulated the expression of Bax and GSDME-N in a xenograft mouse model, with a reduction in tumor size. Our results revealed that CC-115 suppresses tumor growth by inducing GSDME-mediated pyroptosis through the Akt/Bax-mitochondrial intrinsic pathway, indicating CC-115 as a promising therapeutic agent for LUAD.

## Introduction

According to the latest global cancer statistics, lung cancer remains the leading cause of cancer-related deaths, accounting for 18% of deaths of all patients with cancer worldwide. In China, 19% of cancer-related mortality is attributable to lung cancer 1, which is perhaps due to the increasing number of patients with lung cancer being young smokers 2. According to histopathological staging, 85% of lung cancers are non-small cell lung cancer, of which lung adenocarcinoma (LUAD) is the most common subtype (50% of cases) 3. Currently, the early diagnosis rate of lung cancer in China is very low, and the 5-year survival rate is only about 15.6%, mainly because approximately 75% of patients have an advanced disease at the time of diagnosis 4, 5. Chemotherapy is the first-line treatment for advanced lung cancer. However, with the widespread use of chemotherapeutic drugs, more than 60% of patients develop chemoresistance, resulting in poor chemotherapeutic efficacy [6‒8]. Therefore, exploring novel chemotherapeutic agents to overcome chemoresistant environments is key to improving the poor prognosis of patients with LUAD.

Strategies to overcome chemoresistance in cancer include three main aspects: mRNA modification, non-coding RNA modification, and post-translational modification of molecules, including drug target modification and cell death resistance 9-11. Regarding cell death mode, numerous studies have shown that pyroptosis can influence cancer development and play an important role in cancer chemotherapy 12, 13. Pyroptosis is a type of programmed cell death that is distinct from apoptosis and necrosis. It is characterized by swelling of cells and the appearance of large bubbles on the plasma membrane 14-16. Pyroptosis can be divided into two major types: in one type, caspase-1 activation cleaves gasdermin D (GSDMD) into a GSDMD-N fragment 14, 17, and in the other type, caspase-3 activation cleaves gasdermin E (GSDME) into a GSDME-N fragment, both with pore-forming activity [17‒19]. The GSDME-N fragment, which recognizes and binds to cardiolipin and phosphatidylinositol, forms a large number of pores and thus triggers cell pyroptosis 20, 21. This mechanism is one of the major causes of cell death after chemotherapy [22‒24].

The Pyroptosis Compound Library (Selleck Chemicals, Houston, TX, USA) of 441 compounds has been used for high-throughput screening of potential novel chemotherapeutic agents. The library includes compounds with diverse structures, high purity, pharmaceutical activity, cell permeability, and a modulating effect on pyroptosis, some of which are Food and Drug Administration-approved compounds. Our previous experiments at the cellular level revealed that CC-115, among these compounds, exhibits the strongest cytotoxic effect on LUAD cells. CC-115 is a dual inhibitor of the DNA-dependent protein kinase (DNA-PK) and the mammalian target of rapamycin (mTOR) 25. The *in vitro* antitumor effects of CC-115 have been demonstrated in breast and renal cancers as well as in hematologic cancers [26‒28].

Importantly, CC-115 has good physicochemical and pharmacokinetic properties and safe *in vitro* and *in vivo* profiles. Additionally, CC-115 activates the *in vivo* mTOR pathway and inhibits tumor growth [29‒31]. Other PI3K/mTOR inhibitors such as LY3023414 [32‒35] and voxtalisib (SAR245409, XL765) [36‒38], in combination with standard chemotherapeutic agents, have shown efficient antitumor effects in animal models and human xenografts, respectively. Therefore, we speculate that CC-115 has similar effects in LUAD. In this study, we aimed to clarify the molecular mechanism by which CC-115 induces pyroptosis of LUAD cells and demonstrate its potential as a novel chemotherapeutic agent for this disease.

## Materials and Methods

### Reagents

Primary antibodies against DNA-PK (19983-1-AP), mTOR (28273-1-AP), p-mTOR (67778-1-Ig), S6K1 (14485-1-AP), Akt (10176-2-AP), p-Akt (66444-1-Ig), Bax (50599-2-Ig), cytochrome c (10993-1-AP), and GAPDH (60004-1-Ig), as well as secondary antibodies, were obtained from Proteintech Group, Inc. (Wuhan, China). Primary antibodies against caspase-1 (ab179515), GSDMD (ab219800), GSDMD-N (ab215203), caspase-3 (ab32351), GSDME and GSDME-N (ab215191), and p-DNA-PK (ab124918) were purchased from Abcam (Cambridge, MA, USA). Anti-γH2AX (9718) and anti-p-S6K1 (9205S) were purchased from Cell Signaling Technology (Danvers, MA, USA). The Pyroptosis Compound Library (L7400), CC-115 (S7891), and the Akt activator SC79 (S7863) were purchased from Selleck Chemicals (Houston, TX, USA).

### Cell Culture

Human LUAD cell lines (A549 and H1650) were purchased from the American Type Culture Collection (Manassas, VA, USA) and authenticated using STR DNA profiling analysis. The STR appraisal reports were issued by Procell Life Science & Technology Co (Wuhan, China). Both cell lines were maintained in RPMI-1640 medium supplemented with 10% fetal bovine serum, 10 mM L-glutamine, and 5 mg/mL penicillin/streptomycin at 37 °C under 5% CO_2_. All media and supplements were purchased from Invitrogen (Carlsbad, CA, USA).

### Cell Viability Assay

The Pyroptosis Compound Library of 441 compounds was stored as 10 mM stock solutions in dimethyl sulfoxide at 4 °C until use. The LUAD cells were seeded in 96-well plates at 5000 cells per well and incubated overnight in a cell incubator at 37 °C under 5% CO_2_. Thereafter, the cells were treated with 10 μM of each compound of the Pyroptosis Compound Library for 24 h and cell viability was measured using a Cell Counting Kit-8 (CCK-8; Beyotime Biotech, Beijing, China); the absorbance of the samples was recorded at 450 nm.

### Colony Formation Assay

Briefly, A549 cells were seeded in 60-mm plates at a density of 500 cells per well after treatment with CC-115 at different concentrations. Thereafter, the cells were cultured for 10 days, with medium renewal every 3 days. Finally, the colonies were washed with PBS, stained with 0.05% crystal violet, and manually counted.

### BrdU ELISA Assay

Following treatment with CC-115, a BrdU enzyme-linked immunosorbent assay (ELISA) kit (Beyotime Biotech, Beijing, China) was used to determine cell growth. The absorbance of the samples was recorded at 450 nm.

### Western Blotting

Western blot assays were performed as previously described 7, 39. For signal development, the blotted proteins were conjugated with chemiluminescent horseradish peroxidase substrate (MilliporeSigma, Burlington, MA, USA), visualized, and quantified using Quantity (ver 5.2; Bio-Rad Laboratories, Hercules, CA, USA), according to the manufacturer's instructions.

### Total RNA Extraction and qRT-PCR

The total RNA was extracted using a Total RNA Extraction Kit (Solarbio, Beijing, China), according to the manufacturer's instructions, and reverse transcribed using an iScript cDNA Synthesis Kit (Bio-Rad Laboratories) 39. Next, qRT-PCR was performed using a CFX96 Real-time System (Bio-Rad Laboratories) with SYBR Green Supermix (Bio-Rad Laboratories). Both steps were performed according to the manufacturer's instructions. *β-Actin* was used as the internal control for qRT-PCR analysis. The sequences of the primers used in this study were as follows: *GSDME-F*, ATGTTTGCCAAAGCAACCAGGA; *GSDME-R*, TCATGAATGTTCTCTGCCTAAAGCACA; *Bax-F*, CTCAGGATGCGTCCACCAAGAAG; *Bax-R*, CTGTGTCCACGGCGGCAATC; *β-actin-F*, CCTGGCACCCAGCACAAT; *β-actin-R*, GGGCCGGACTCGTCATAC.

### GSDME Knockdown and Bax Knockout

For *GSDME* knockdown, a short hairpin RNA (shRNA) targeting the si-h-coding sequences was designed and inserted into a pGMLV-SC5 RNAi lentiviral vector. The interference target sequences were as follows: *NC*: TTCTCCGAACGTGTCACGT; *H_GSDME-shRNA1*: GATGATGGAGTATCTGATCTT; *H_GSDME-shRNA2*: GGATTGTGCAGCGCTTGTTTG; *H_GSDME-shRNA3*: GCTTTAGGCAGAGAACATTCA. *GSDME* knockdown was performed by Genomeditech (Shanghai, China). The knockdown efficiency was evaluated using qRT-PCR and western blotting. Cells were transfected using Lipofectamine 3000 (Invitrogen) and harvested after 48 h for further experiments. To generate *Bax*-knockout cells, a pair of short guide-RNA was produced by RiboBio Co., Ltd. (Guangzhou, China): forward: 5′-CACCGGTTTCATCCAGGATCGAGCA-3′; reverse: 5′-AAACTGCTCGATCCTGGATGAAACC-3′. BAX short guide-RNA plasmids were then transfected into A549 and H1650 cells using Lipofectamine 3000 (Invitrogen). The knockout efficiency was evaluated using qRT-PCR and western blotting.

### Immunofluorescence and Immunohistochemistry

Immunofluorescence 39 and immunohistochemistry 7 assays were performed as previously reported. In both cases, images were acquired using an Olympus BX51 microscope (Olympus Corporation, Tokyo, Japan) equipped with various objective lenses (10×, 20×, and 40×) and a DP50 camera. Images were processed using DPC controller software (Olympus Corporation).

### Lactase Dehydrogenase (LDH) and IL-1β Release Assay

LDH release was tested using an LDH Cytotoxicity Assay Kit (Beyotime Biotech). The level of IL-1β was measured using a QuantiCyto IL-1β ELISA Kit (Enzyme-linked Biotechnology, Shanghai, China), according to the manufacturer's instructions. The absorbance of the samples was measured at 450 nm.

### Flow Cytometry

An Annexin V-PE/7-AAD Detection Kit (KeyGEN, Jiangsu, China) was used to measure pyroptosis using flow cytometry, according to the manufacturer's instructions. A flow cytometer (FACSCalibur, BD Biosciences, Franklin Lakes, NJ, USA) and its software FlowJo were used to analyze the cells.

### Mitochondrial Membrane Potential Assay

A JC-10 Mitochondrial Membrane Potential Assay Kit (Solarbio) was used to assess the changes in mitochondrial membrane potential in A549 and H1650 cells after treatment with CC-115 and/or SC79 for 48 h, according to the manufacturer's instructions. The cells were stained with JC-10 working solution for 15 min at 37 °C, washed twice with JC-10 staining buffer, and observed under an Olympus BX51 microscope (Olympus Corporation). The JC-10 aggregate to monomer ratio was analyzed using DPC controller software (Olympus Corporation).

### Xenograft Mouse Model

All xenograft experiments were performed following the guidelines of the Laboratory Animal Ethical Committee of Chengdu Medical College. All experimental protocols were approved by the Laboratory Animal Ethical Committee at Chengdu Medical College. A subcutaneous xenograft mouse model of LUAD was established to explore the therapeutic potential of CC-115 on LUAD *in vivo*. Female *BALB/C* nude mice (4-5 weeks of age, 14-16 g) were purchased from Dossy Experimental Animals Co., Ltd. (Chengdu, China). Before conducting the experiments, the mice were randomly assigned to four treatment groups of five mice each: normal control (NC), CC-115, NC + si-GSDNE, and CC-115 + si-GSDNE. A549 cells (1 × 10^6^) with or without *GSDME* knockdown were suspended in 100 μL of serum-free RPMI-1640 medium and injected subcutaneously into the axilla of nude mice. Seven days after cell implantation, when the tumor volume was approximately 30-40 mm^3^, the mice in each group were intraperitoneally injected with 100 μL of PBS or CC-115 (2.5 mg/kg per day). Tumor growth was measured using vernier calipers every 7 days, and tumor volume was determined using the formula V = (*a* × *b*^2^)/2, where *a* and *b* are the maximum and minimum diameters in millimeters, respectively. After 3 weeks, the mice were sacrificed and weighed immediately after dissection.

### Statistical Analysis

Data analysis was performed using GraphPad Prism v. 7.0 (GraphPad Software, San Diego, CA, USA). All *in vitro* experiments were independently performed at least three times. The results are presented as mean ± standard deviation unless otherwise indicated. The significance of difference between experimental groups was determined using the two-tailed Student's* t*-test. Mann-Whitney *U-*test was used for data that did not conform to a normal distribution. *P* < 0.05 indicated significance.

## Results

### CC-115 Inhibits LUAD Cell Survival Via Dual Inhibition of DNA-PK and mTOR

From the 441 Pyroptosis Compound Library, we selected the 20 most lethal ones to LUAD cells (Figure S1A). The lethality of these 20 compounds was further confirmed via secondary screening using the CCK-8 assay (Figure S1B). The results showed that CC-115 (C16H16N8O) was the most potent compound inhibiting LUAD cell viability (Figure S1C).

**Figure 1:**
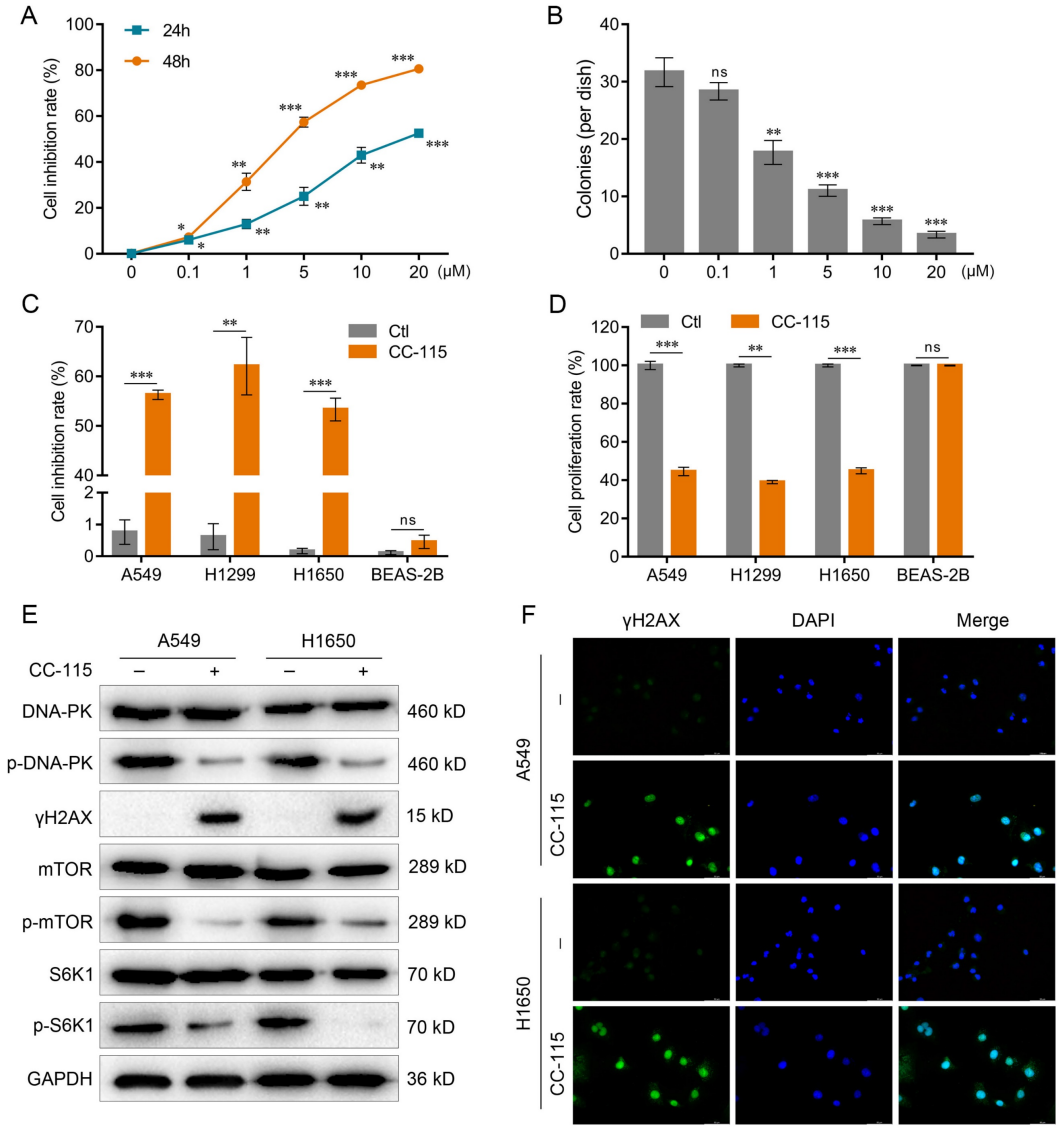
CC-115 inhibits LUAD cell survival via the dual inhibition of DNA-PK and mTOR. (**A**) Results of the CCK-8 assay. A549 cells were treated with CC-115 of different concentrations for the indicated periods. (**B**) Colony formation assay in A549 cells with different concentrations of CC-115. (**C**) The CCK-8 assay in the indicated cell lines. (**D**) Bar graph showing the BrdU ELISA assay for determining the proliferation of the indicated cell lines. (**E**) Western blotting of DNA-PK and mTOR pathway markers in LUAD cells. (**F**) Immunofluorescence staining of γH2AX in LUAD cells with or without CC-115 treatment (scale bar, 50 μm). *P < 0.05, **P < 0.01, ***P < 0.001, ns: non-significant. Ctl: control.

To further explore this effect, we gradually increased CC-115 concentration. The CCK-8 assay was performed to observe the induction of cell death by CC-115 after 24-48 h; the colony formation assay was performed after 48 h of treatment. These assays indicated that 5 μM was the most lethal concentration (Figure 1A, B), as observed by a significant reduction in the viability and inhibition of proliferation of LUAD cells after 48 h of treatment (A549, H1299, and H1650; Figure 1C, D). Previous studies have shown that CC-115 acts as a dual DNA-PK/mTOR inhibitor 27, 28. Therefore, we evaluated if its inhibitory effect on LUAD cell viability might be through DNA-PK/mTOR pathway. Our results showed that CC-115 could reduce DNA-PK activity, increase the expression of the DNA-damage marker γH2AX, and decrease the expression of the mTOR pathway markers p-mTOR and p-S6K1 in LUAD cells (Figure 1E, F). Collectively, our results show that CC-115 can block DNA-PK and mTOR activation and inhibit LUAD cell survival.

### CC-115 Induces Pyroptosis of LUAD Cells

**Figure 2:**
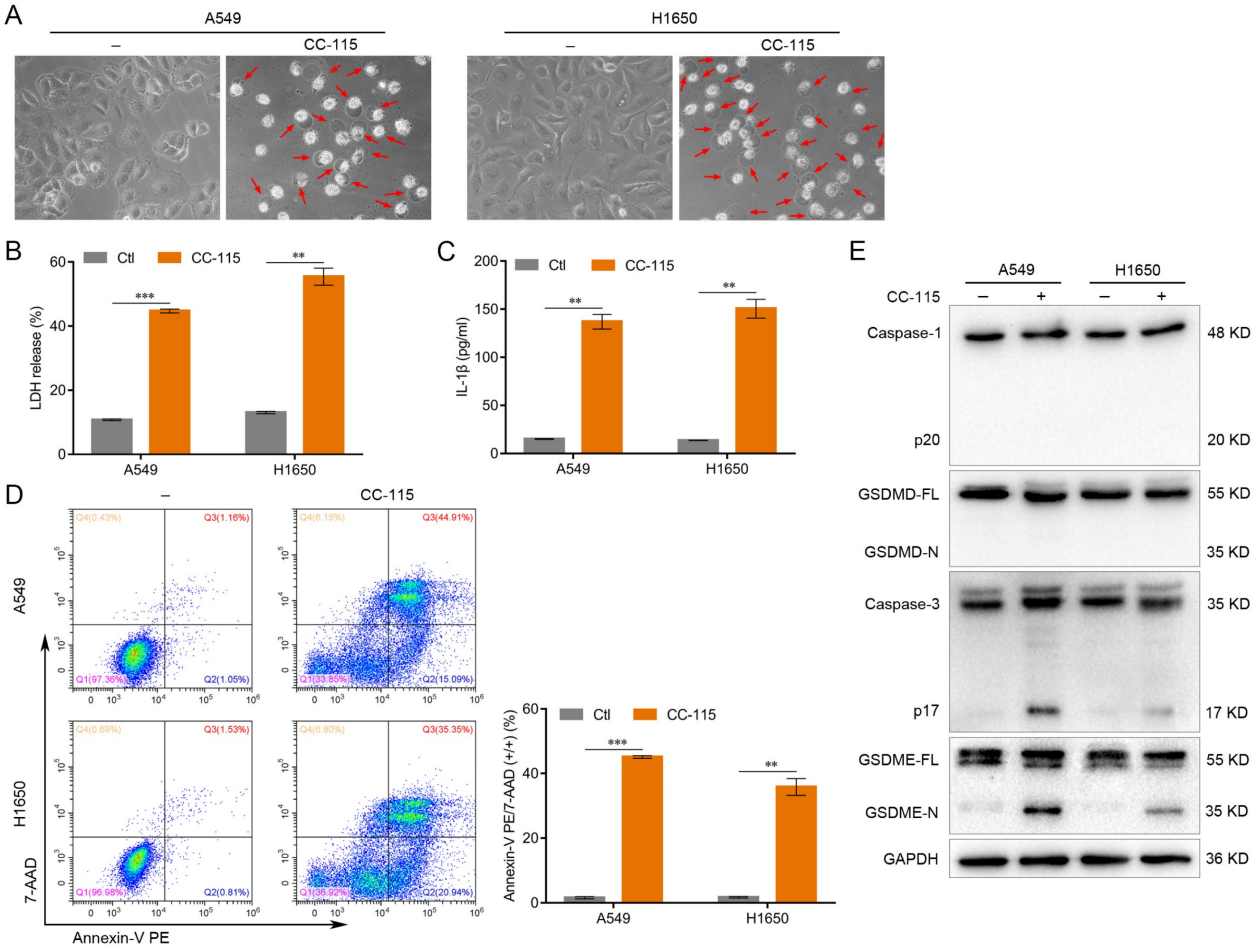
CC-115 induces the pyroptosis of LUAD cells. (**A**) Representative images of LUAD cells treated with CC-115 (scale bar, 50 μm). (**B, C**) Results of the ELISA assay used to quantify LDH release and IL-1β secretion in LUAD cells. (**D**) Flow cytometry plots showing the percentage of Annexin V PE and 7-AAD double-positive LUAD cells. (**E**) Western blotting of pyroptosis markers in LUAD cells treated with CC-115. **P < 0.01, ***P < 0.001. Ctl: control.

To investigate the effect of CC-115 on LUAD cell pyroptosis, we treated A549 and H1650 cells with CC-115 (5 μM) for 48 h. Morphologically, CC-115-treated A549 and H1650 cells exhibited swelling and large bubbles (Figure 2A), which closely resembled the characteristics of cell pyroptosis 12. In addition, CC-115 promoted the release of LDH and the secretion of IL-1β in both cell lines (Figure 2B, C). Additionally, the flow cytometry analysis showed a higher number of Annexin V-PE and 7-AAD double-positive cells under CC-115 treatment than in the control (Figure 2D). Pyroptosis is triggered by the N-terminal domain of the gasdermin family members, of which the two most important members are GSDMD, cleaved by caspase-1, and GSDME, cleaved by caspase-3 15, 19. Active caspase-1 and GSDMD cleavage were not observed in CC-115-treated A549 and H1650 cells (Figure 2E). However, CC-115 treatment led to significant levels of both active caspase-3 and N-terminal fragment of GSDME (GSDME-N) in both A549 and H1650 cells (Figure 2E). These results indicate that GSDME rather than GSDMD is cleaved during CC-115-induced pyroptosis in LUAD cells.

### GSDME Knockdown Abrogates the Pyroptosis-Inducing Effect of CC-115 in LUAD Cells

**Figure 3:**
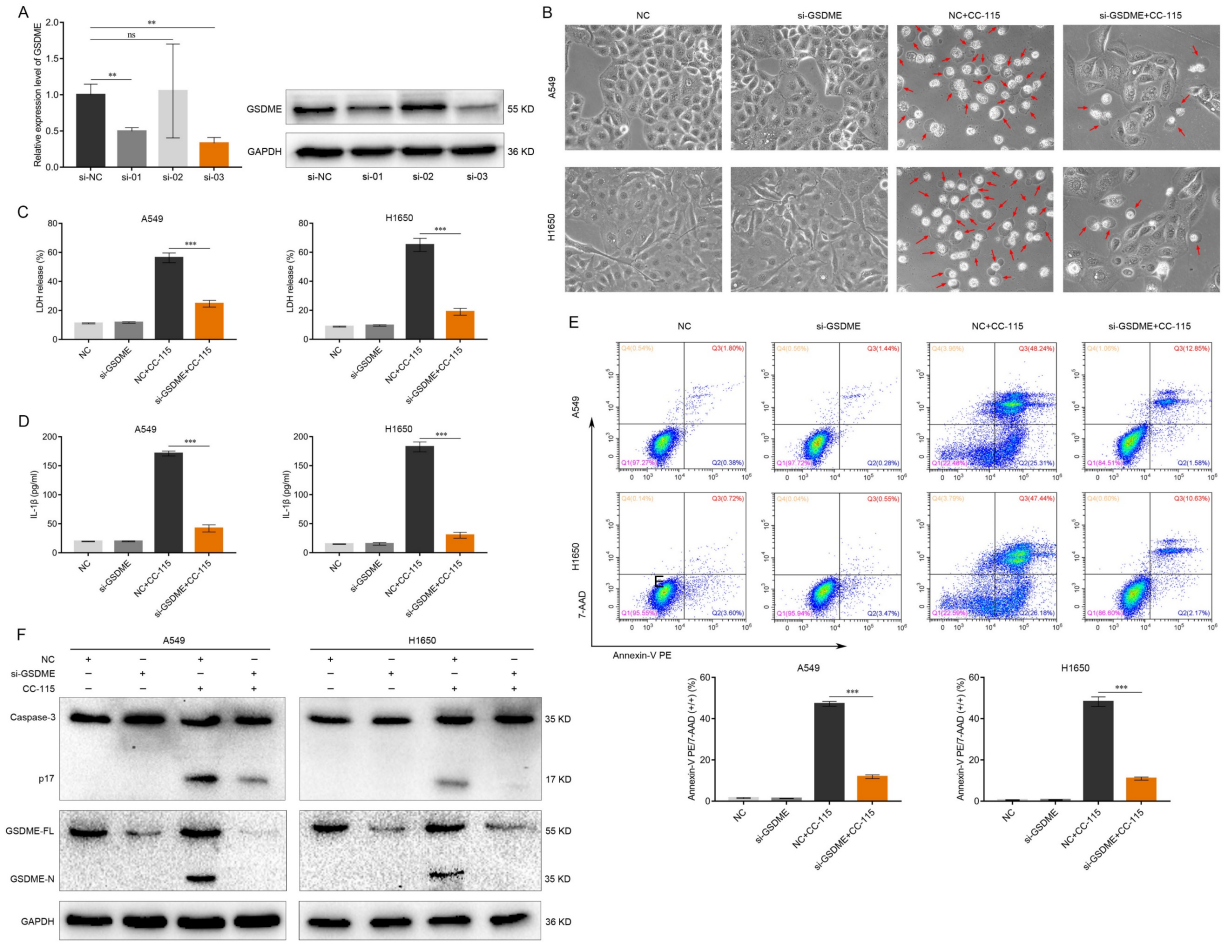
*GSDME* knockdown abrogates the pyroptosis-inducing effect of CC-115 in LUAD cells. (**A**) Knockdown efficiency of GSDME determined via qRT-PCR and western blotting. (**B**) Representative images of LUAD cells in the indicated groups (scale bar, 50 μm). (**C, D**) ELISA determination of the release of LDH and secretion of IL-1β in the indicated groups. (**E**) Flow cytometry plots showing the percentage of Annexin V PE and 7-AAD double-positive cells in the indicated groups. (**F**) Western blotting of pyroptosis markers in the indicated groups. **P < 0.01, ***P < 0.001, ns: not significant.

To investigate whether GSDME is essential for CC-115-induced pyroptosis of LUAD cells, an shRNA lentiviral vector was used to stably knockdown *GSDME* in A549 and H1650 cells. As shown in Figure 3A, the mRNA and protein levels of GSDME were reduced by at least 65% by *H_GSDME-shRNA3*. Therefore, we selected this shRNA for the subsequent experiments. *GSDME* knockdown significantly decreased the amount of cell swelling and large bubbles induced by CC-115 in A549 and H1650 cells (Figure 3B). Moreover, *GSDME* knockdown attenuated the release of LDH and secretion of IL-1β (Figure 3C, D), and reduced the number of annexin V-PE and 7-AAD double-positive LUAD cells (Figure 3E). Finally, the increased levels of active caspase-3 and GSDME-N were reversed by *GSDME* knockdown (Figure 3F), suggesting that CC-115 potentially inhibits LUAD cell survival via GSDME-mediated pyroptosis.

### CC-115 Regulates GSDME-Dependent Pyroptosis through Akt/Bax Signaling

**Figure 4:**
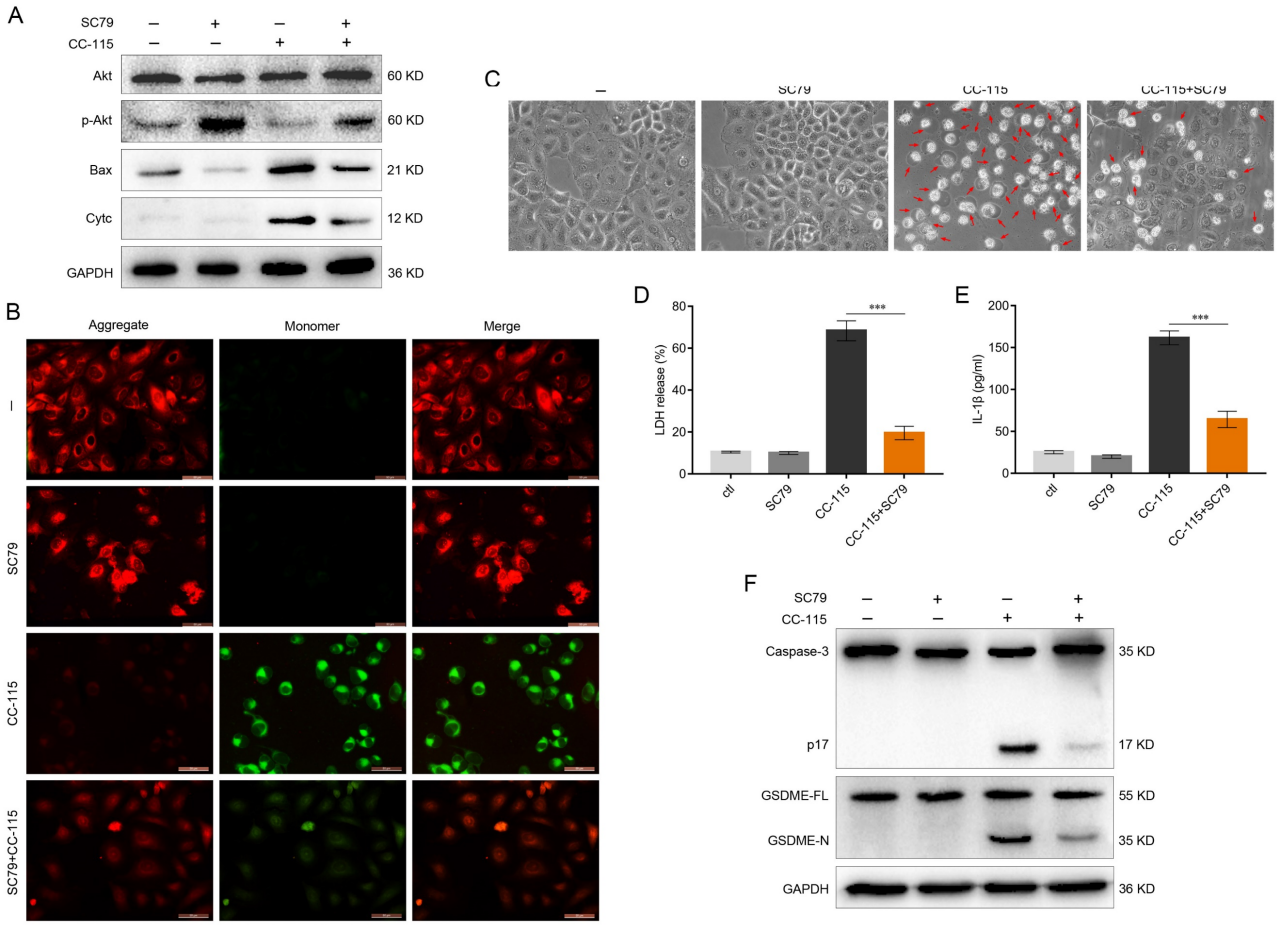
CC-115 regulates GSDME-dependent pyroptosis through Akt/Bax signaling. (**A**) Western blotting of Akt, Bax, and Cyt-c in A549 cells treated with or without CC-115 (5 μM) and/or SC79 (5 μM). (**B**) Representative images of the mitochondrial membrane potential signal in A549 cells determined using the JC-10 assay in the indicated groups (scale bar, 50 μm). (**C**) Representative bright field images of A549 cells in the indicated groups (scale bar, 50 μm). (**D, E**) Release of LDH and secretion of IL-1β in A549 cells as determined using ELISA. (**F**) Analysis of caspase-3 and GSDME expression using western blotting in A549 cells in the indicated groups. ***P < 0.001.

We explored the potential molecular mechanism by which CC-115 might trigger GSDME-dependent pyroptosis. DNA-PK is a key molecule in the DNA-damage response and plays an important role in detecting and repairing DNA double-strand breaks by non-homologous end-joining 40. mTOR, a member of the PI3K protein kinase family, is targeted by specific inhibitors that not only inhibit mTOR activity but also directly inhibit Akt activity 41. Based on the Kyoto Encyclopedia of Genes and Genomes pathway analysis, we found that both DNA-PK and mTOR can promote the activation of Akt. It has been suggested that Akt could inhibit the expression of Bax through the mitochondrial intrinsic pathway, thereby suppressing the onset of apoptosis 42. Interestingly, it has been reported that the mitochondrial intrinsic pathway can also mediate cell pyroptosis via the Bax-caspase-3-GSDME pathway 43. We found that CC-115 significantly inhibited Akt phosphorylation in A549 and H1650 cells, an effect reversed by the Akt activator SC79 (Figures 4A, S2A). In addition, the effects of CC-115 on Bax level and cytochrome c release were reduced by treatment with SC79 (Figures 4A, S2A). Next, to determine the changes in the mitochondrial membrane potential of LUAD cells after CC-115 treatment, we used JC-10 staining. Notably, CC-115 treatment significantly decreased the ratio of JC-10 aggregates (red) to monomers (green), indicating a reduction in membrane potential in both cell lines after treatment (Figures 4B, S2B). Interestingly, the SC79 Akt activator reversed such an effect (Figures 4B, S2B). Consistent with these results, we observed that SC79 significantly suppressed the CC-115-induced cell swelling, the release of LDH and secretion of IL-1β, and the expression of active-caspase-3 and GSDME-N (Figures 4C-F, S2C-F). These results indicate that CC-115 induces GSDME-dependent pyroptosis through Akt/Bax signaling.

### GSDME-Dependent Pyroptosis Induced by CC-115 is Downstream of the Bax-Mitochondrial Intrinsic Pathway

**Figure 5:**
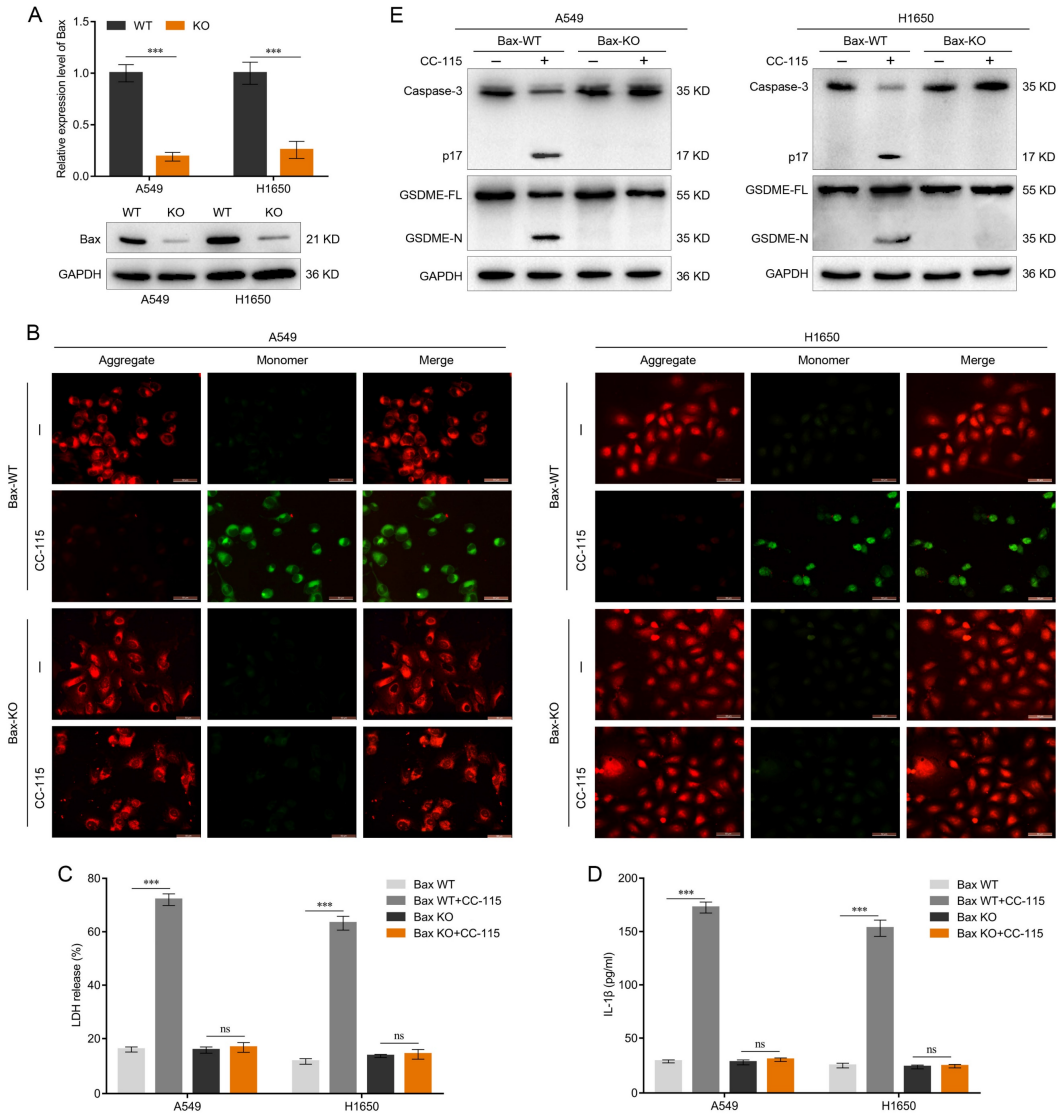
CC-115-induced GSDME-dependent pyroptosis is downstream of the Bax-mitochondrial intrinsic pathway. (**A**) Knockout efficiency of *Bax* determined via qRT-PCR and western blotting. (**B**) Representative images of the mitochondrial membrane potential of LUAD cells determined using the JC-10 assay in the indicated groups (scale bar, 50 μm). (**C, D**) Release of LDH and secretion of IL-1β in LUAD cells in the indicated groups. (**E**) Western blotting of GSDME-dependent pyroptosis markers in LUAD cells in the indicated groups. ***P < 0.001, ns: not significant.

To further elucidate the role of Bax in CC-115-mediated pyroptosis, we generated *Bax*-knockout LUAD cells. qRT-PCR and western blotting were performed to determine the knockout efficiency of *Bax* in A549 and H1650 cells (Figure 5A). In *Bax* wild-type (WT) cells, CC-115 treatment reduced JC-10 staining, suggesting decreased mitochondrial membrane potential (Figure 5B). In contrast, *Bax* knockout abrogated the effect of CC-115 on mitochondrial membrane potential (Figure 5B) and the release of LDH and secretion of IL-1β induced by CC-115 treatment (Figure 5C, D) in A549 and H1650 cells. As caspase-3 activation is downstream of the Bax-mitochondrial intrinsic pathway 21,43, we determined caspase-3 activity in *Bax*-knockout cells. Our results showed that CC-115-induced caspase-3 activity was considerably inhibited in A549 and H1650 *Bax*-knockout cells (Figure 5E). As expected, western blotting showed decreased levels of the N-terminal fragment of GSDME in *Bax*-knockout cells and inhibition of GSDME-N generation induced by CC-115 treatment (Figure 5E). Thus, we conclude that the Bax-mitochondrial intrinsic pathway is essential for CC-115-induced GSDME-dependent pyroptosis in LUAD.

### CC-115 Exerts an Antitumor Effect through GSDME-Dependent Pyroptosis *In vivo*

**Figure 6:**
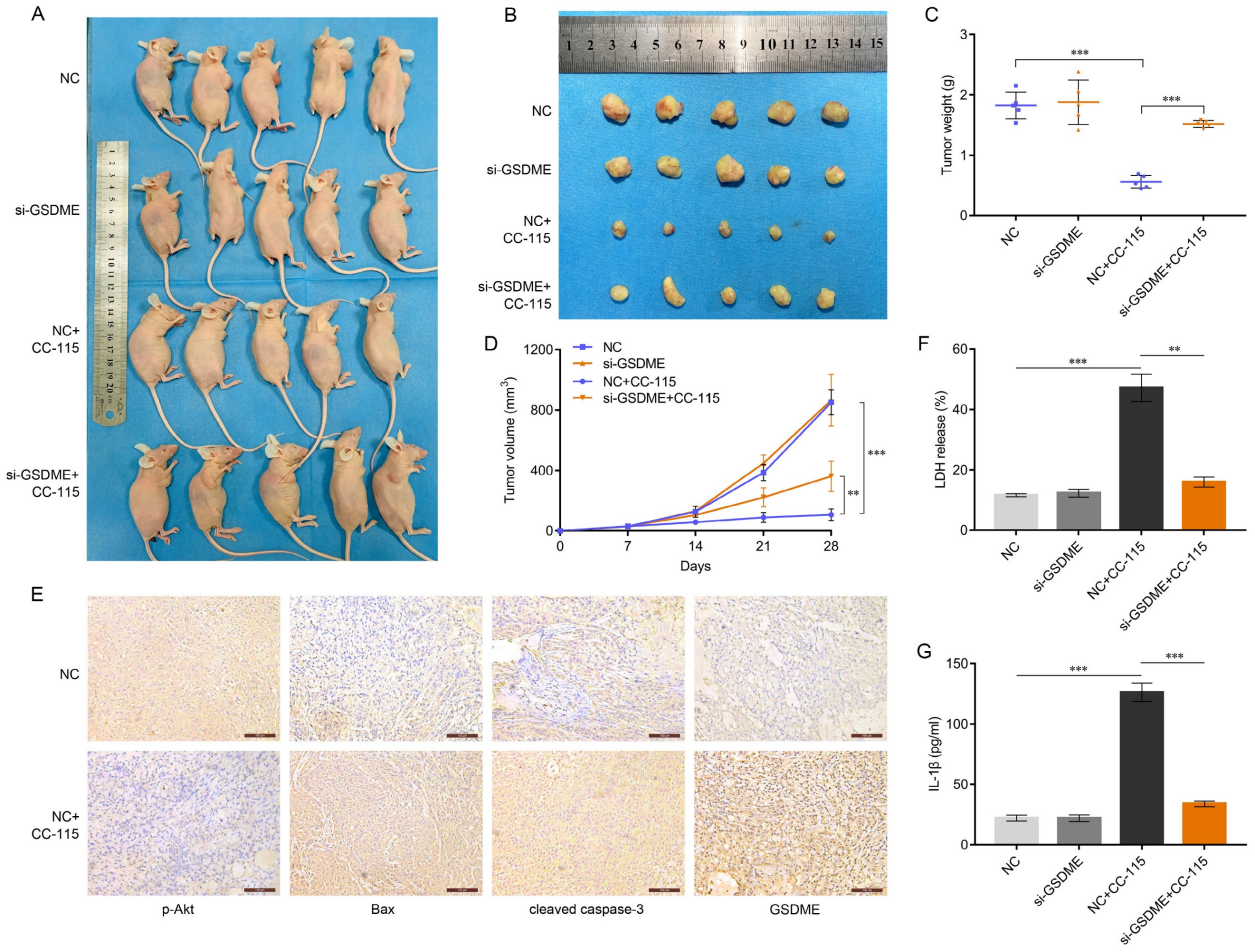
CC-115 shows the antitumor effect through GSDME-dependent pyroptosis *in vivo*. (**A**) Images of nude mice with subcutaneous xenografts in the indicated groups. (**B**) Xenograft tumors isolated from sacrificed mice (28 days) with or without CC-115 treatment (2.5 mg/kg per day). (**C**) Average weights of xenograft tumors in the indicated groups. (**D**) Kinetics of tumor growth by volume of subcutaneous xenograft tumors. (**E**) Immunohistochemistry of p-Akt, Bax, cleaved caspase 3, and GSDME in subcutaneous mouse tumors from the NC vs. NC+CC-115 groups (scale bar, 100 μm). (**F, G**) Release of serum LDH and secretion of IL-1β in nude mice. **P < 0.01, ***P < 0.001.

As our *in vitro* results suggested that CC-115 induces GSDME-dependent pyroptosis through the Akt/Bax pathway, we explored the therapeutic potential of CC-115 *in vivo*. We established a xenograft model on *BALB/C* nude mice using WT or *GSDME*-knockdown A549 cells (Figure 6A). Although the tumor weight and volume were comparable between the *GSDME*-knockdown (si-GSDME) and normal control (NC) groups (Figure 6B-D), CC-115 treatment significantly reduced tumor weight and volume. As expected, *GSDME* knockdown reduced the antitumor effect of CC-115, as tumor size reduction was not very significant (Figure 6B-D). Immunohistochemistry analysis of the tumors showed that CC-115 induced Bax, cleaved caspase-3, and GSDME expression but reduced the level of p-Akt (Figure 6E). Furthermore, CC-115 promoted the release of LDH and secretion of IL-1β, but *GSDME* knockdown significantly attenuated this effect (Figure 6F, G). These results emphasize that CC-115 inhibits tumor growth *in vivo* by inducing GSDME-dependent pyroptosis.

## Discussion

Chemotherapeutic agents remain the first-line treatment for many solid tumors, including lung and breast cancers. However, the development of drug resistance is a major cause of treatment failure [7, 44‒46]. Therefore, exploring novel chemotherapeutic agents to counteract the development of a drug-resistant environment is essential to achieve therapeutic success. Recent strategies to overcome chemoresistance in cancer include three main aspects: mRNA modification, non-coding RNA modification, and post-translational modification of molecules, including drug target modification and cell death resistance 9-11. Furthermore, cell death resistance is a hallmark of cancer cells 47, 48. There are various mechanisms of cell death, including apoptosis and autophagy, evasion of which is among the main causes of chemoresistance in cancer 12, 49-52. Cell pyroptosis is another form of cell death and is also one of the main causes of cell resistance after chemotherapy 53, 54. Therefore, we selected The Pyroptosis Compound Library to find a potential compound for LUAD chemotherapy.

The Pyroptosis Compound Library is a unique collection of 441 pyroptosis-related compounds that are structurally diverse, pharmaceutically active, cell-permeable, and some are FDA-approved, with potential application in new drug development and overcoming drug resistance 55. Among these compounds, we demonstrated that CC-115 has the strongest killing effect on LUAD cells (Figure S1); therefore, we investigated the molecular mechanisms underlying the anticancer effect of CC-115 on LUAD cells. Previous studies have shown that CC-115 is a dual inhibitor of DNA-PK and mTOR and effective against breast and renal cancers 25, 26. Consistent with the findings of these studies, we found that CC-115 significantly inhibited the viability of LUAD cells by reducing DNA-PK activity, increasing the expression of the DNA-damage marker γH2AX, and decreasing the expression of the mTOR pathway markers p-mTOR and p-S6K1 (Figure 1). These results indicate that CC-115 can block DNA-PK and mTOR activation and inhibit the survival of LUAD cells.

It is well known that cancer cell death consists mainly of apoptosis, necrosis, autophagy and pyroptosis 12-16. Apoptosis is characterized morphologically by nuclear pyknosis and the formation of apoptotic body, with the cell membrane remaining intact. In contrast, pyroptosis is a continuous swelling of the cell, leading to the rupture of the cell membrane and the release of the cell contents 17, 20. In our study, we found that after CC-115 treatment of lung adenocarcinoma cells resulted in swelling and large bubbles with cell membrane rupture (Figure 2A), which closely resembled the characteristics of pyroptosis. Moreover, recent studies have revealed a switch between apoptosis and pyroptosis, in which caspase-3 and GSDME play a key role 12. When GSDME is highly expressed, chemotherapeutic agents induce tumor cell death through caspase-3-dependent pyroptosis. When GSDME expression is low, the cell death mode shifts to apoptosis 12, 56. In our study, we found that GSDME was highly expressed in lung adenocarcinoma cells (Figure 2E). Therefore, we conducted additional experiments to further explore whether CC-115 exerts anticancer effects through pyroptosis. We found that CC-115 could inhibit LUAD progression by regulating the onset of pyroptosis mediated by GSDME (Figures 2, 3). This finding suggests that CC-115 may be a potential drug with improved therapeutic efficacy.

Based on the Kyoto Encyclopedia of Genes and Genomes pathway analysis, we found that both DNA-PK and mTOR can promote the activation of Akt. Research has shown that Akt can disturb the BCL-2/Bax balance and inhibit cytochrome c release, suppressing apoptosis 57, 58. Interestingly, it has been suggested that the mitochondrial intrinsic pathway can also mediate cell pyroptosis via Bax-caspase-3-GSDME 59, 60. In the present study, we found that CC-115 can significantly inhibit the phosphorylation of Akt and suppress the inhibitory effect of Akt on Bax, thus activating the mitochondrial intrinsic pathway and inducing pyroptosis via Bax-caspase-3-GSDME (Figures 4, S2). It was expected that SC79, an Akt activator, would reverse the effects of CC-115. Similarly, *Bax* knockout abrogated the effect of CC-115 on mitochondrial membrane potential and suppressed the CC-155-mediated increase in caspase-3 activity and GSDME-N expression, thereby inhibiting the onset of GSDME-dependent pyroptosis (Figure 5). Consequently, our study demonstrates that CC-115-induced GSDME-dependent pyroptosis is downstream of the Akt/Bax-mitochondrial intrinsic pathway.

Finally, to determine the effect of CC-115 on LUAD *in vivo*, we established a subcutaneous tumor xenograft mouse model. We demonstrated that CC-115 can significantly reduce tumor size, and that this effect is due to the induction of Bax and pyroptosis markers, and the suppression of Akt phosphorylation (Figure 6). These results are supported by the fact that *GSDME* knockdown attenuated the effects of CC-115 on Bax/caspase-3/GSDME-mediated pyroptosis *in vivo*. However, whether combination therapy enhances the efficacy of CC-115 remains to be explored; studies should also focus on the potential mechanism of secondary pyroptosis.

## Conclusions

**Figure 7:**
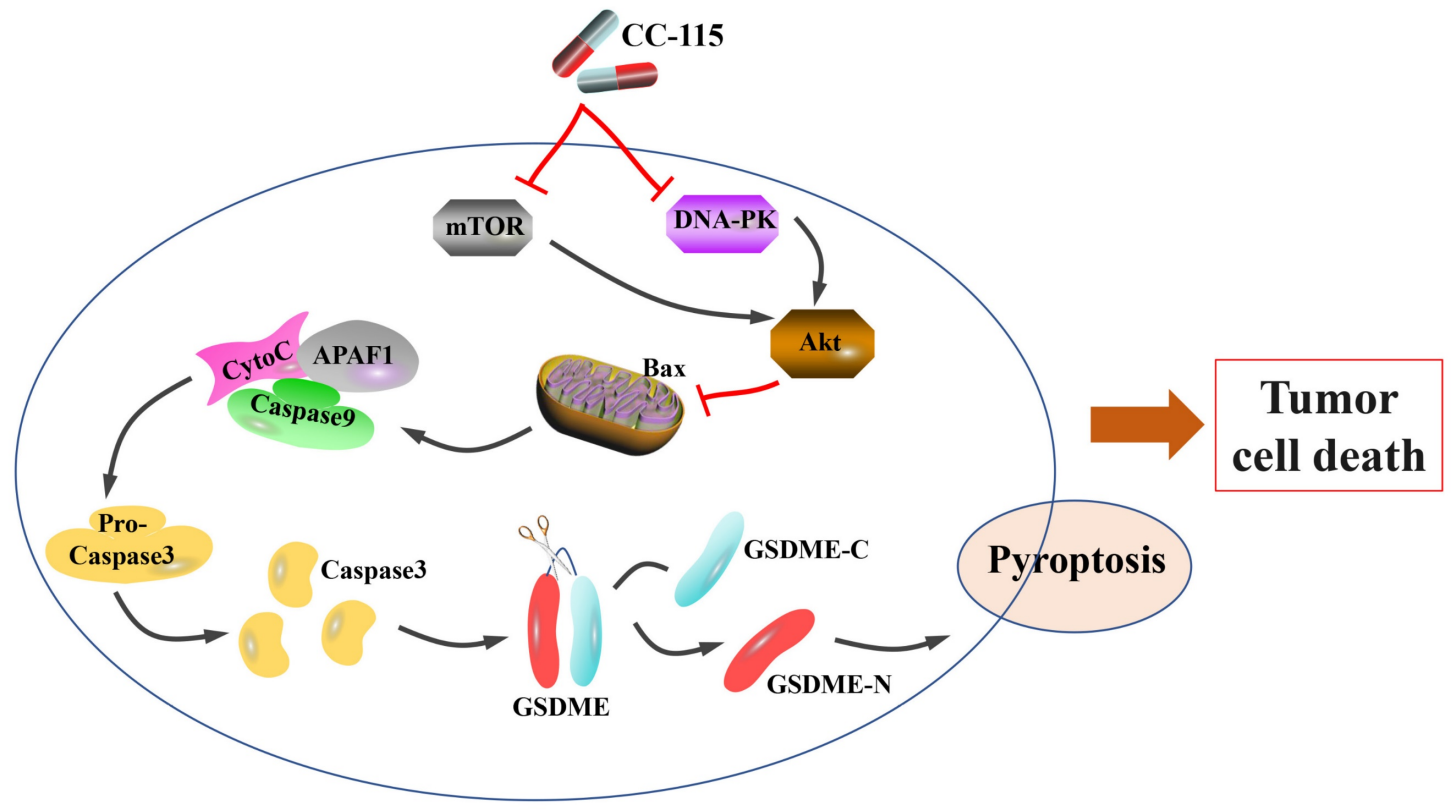
Diagram of the proposed mechanism and function of CC-115 in LUAD. CC-115 exerts its function as a dual DNA-PK/mTOR inhibitor, suppressing the activity of Akt. This induces significant mitochondrial damage and triggers caspase-3 activation, which induces GSDME-dependent pyroptosis, ultimately affecting cell survival in lung adenocarcinoma.

Our results demonstrated the role of CC-115 in mediating GSDME-dependent pyroptosis *in vitro* and *in vivo* to regulate LUAD progression. In summary, we demonstrated that CC-115 is a dual inhibitor of DNA-PK and mTOR, suppressing Akt activity and therefore, its effect on *Bax*. This effect induces significant mitochondrial damage, triggering caspase-3 activation, which in turn induces GSDME-dependent pyroptosis, ultimately affecting cell survival in LUAD (Figure 7). In conclusion, CC-115 is a potential drug to improve the effectiveness of LUAD chemotherapy.

## Supplementary Material

Supplementary figures.

## Abbreviations

LUAD: lung adenocarcinoma
GSDMD: gasdermin D
GSDME: gasdermin E
DNA-PK: DNA-dependent protein kinase
mTOR: mammalian target of rapamycin
PI3K: phosphatidylinositol 3 kinase
CCK-8: cell counting kit-8
PBS: phosphate-buffered saline
ELISA: enzyme-linked immunosorbent assay
LDH: lactate dehydrogenase
IL: interleukin
mRNA: mitochondrial RNA
KEGG: Kyoto Encyclopedia of Genes and Genomes
Akt: protein kinase B

## References

[1] Sung H, Ferlay J, Siegel RL (2021). Global cancer statistics 2020: GLOBOCAN estimates of incidence and mortality worldwide for 36 cancers in 185 countries. CA Cancer J Clin.

[2] Ettinger DS, Wood DE, Aisner DL (2021). NCCN Guidelines insights: Non-small cell lung cancer, version 2.2021. J Natl Compr Canc Netw.

[3] Herbst RS, Heymach JV, Lippman SM (2008). Lung cancer. N Engl J Med.

[4] Cao W, Chen HD, Yu YW (2021). Changing profiles of cancer burden worldwide and in China: a secondary analysis of the global cancer statistics 2020. Chin Med J (Engl).

[5] Wu C, Li M, Meng H (2019). Analysis of status and countermeasures of cancer incidence and mortality in China. Sci China Life Sci.

[6] Cai J, Fang L, Huang Y (2017). Simultaneous overactivation of Wnt/beta-catenin and TGFbeta signalling by miR-128-3p confers chemoresistance-associated metastasis in NSCLC. Nat Commun.

[7] Wu DM, Zhang T, Liu YB (2019). The PAX6-ZEB2 axis promotes metastasis and cisplatin resistance in non-small cell lung cancer through PI3K/AKT signaling. Cell Death Dis.

[8] Kenmotsu H, Yamamoto N, Yamanaka T (2020). Randomized Phase III study of pemetrexed plus cisplatin versus vinorelbine plus cisplatin for completely resected Stage II to IIIA nonsquamous non-small-cell lung cancer. J Clin Oncol.

[9] Holohan C, Van Schaeybroeck S, Longley DB (2013). Cancer drug resistance: an evolving paradigm. Nat Rev Cancer.

[10] Liu K, Gao L, Ma X (2020). Long non-coding RNAs regulate drug resistance in cancer. Mol Cancer.

[11] Song H, Liu D, Dong S (2020). Epitranscriptomics and epiproteomics in cancer drug resistance: therapeutic implications. Signal Transduct Target Ther.

[12] Wang Y, Gao W, Shi X (2017). Chemotherapy drugs induce pyroptosis through caspase-3 cleavage of a gasdermin. Nature.

[13] Yu P, Zhang X, Liu N (2021). Pyroptosis: mechanisms and diseases. Signal Transduct Target Ther.

[14] Lu F, Lan Z, Xin Z (2020). Emerging insights into molecular mechanisms underlying pyroptosis and functions of inflammasomes in diseases. J Cell Physiol.

[15] Man SM, Karki R, Kanneganti TD (2017). Molecular mechanisms and functions of pyroptosis, inflammatory caspases and inflammasomes in infectious diseases. Immunol Rev.

[16] Lamkanfi M, Dixit VM (2014). Mechanisms and functions of inflammasomes. Cell.

[17] Ding J, Wang K, Liu W (2016). Pore-forming activity and structural autoinhibition of the gasdermin family. Nature.

[18] Gou X, Xu W, Liu Y (2022). IL-6 prevents lung macrophage death and lung inflammation injury by inhibiting GSDME- and GSDMD-mediated pyroptosis during pneumococcal pneumosepsis. Microbiol Spectr.

[19] Yao F, Jin Z, Zheng Z (2022). HDAC11 promotes both NLRP3/caspase-1/GSDMD and caspase-3/GSDME pathways causing pyroptosis via ERG in vascular endothelial cells. Cell Death Discov.

[20] Shen X, Wang H, Weng C (2021). Caspase 3/GSDME-dependent pyroptosis contributes to chemotherapy drug-induced nephrotoxicity. Cell Death Dis.

[21] Mai FY, He P, Ye JZ (2019). Caspase-3-mediated GSDME activation contributes to cisplatin- and doxorubicin-induced secondary necrosis in mouse macrophages. Cell Prolif.

[22] Li F, Xia Q, Ren L (2022). GSDME increases chemotherapeutic drug sensitivity by inducing pyroptosis in retinoblastoma cells. Oxid Med Cell Longev.

[23] Wang D, Fu Z, Gao L (2022). Increased IRF9-STAT2 signaling leads to adaptive resistance toward targeted therapy in melanoma by restraining GSDME-dependent pyroptosis. J Invest Dermatol.

[24] Wang S, Zhang MJ, Wu ZZ (2022). GSDME is related to prognosis and response to chemotherapy in oral cancer. J Dent Res.

[25] Bürkel F, Jost T, Hecht M (2020). Dual mTOR/DNA-PK inhibitor CC-115 induces cell death in melanoma cells and has radiosensitizing potential. Int J Mol Sci.

[26] Zheng B, Sun X, Chen XF (2020). Dual inhibition of DNA-PKcs and mTOR by CC-115 potently inhibits human renal cell carcinoma cell growth. Aging.

[27] Tsuji T, Sapinoso LM, Tran T (2017). CC-115, a dual inhibitor of mTOR kinase and DNA-PK, blocks DNA damage repair pathways and selectively inhibits ATM-deficient cell growth *in vitro*. Oncotarget.

[28] Thijssen R, Ter Burg J, Garrick B (2016). Dual TORK/DNA-PK inhibition blocks critical signaling pathways in chronic lymphocytic leukemia. Blood.

[29] Munster P, Mita M, Mahipal A (2019). First-in-human Phase I study of a dual mTOR kinase and DNA-PK inhibitor (CC-115) in advanced malignancy. Cancer Manag Res.

[30] Beebe J, Zhang JT (2019). CC-115, a dual mammalian target of rapamycin/DNA-dependent protein kinase inhibitor in clinical trial, is a substrate of ATP-binding cassette G2, a risk factor for CC-115 resistance. J Pharmacol Exp Ther.

[31] Mortensen DS, Perrin-Ninkovic SM, Shevlin G (2015). Optimization of a series of triazole containing mammalian target of rapamycin (mTOR) kinase inhibitors and the discovery of CC-115. J Med Chem.

[32] Sweeney CJ, Percent IJ, Babu S (2022). Phase Ib/II study of enzalutamide with Samotolisib (LY3023414) or placebo in patients with metastatic castration-resistant prostate cancer. Clin Cancer Res.

[33] Jia N, Che X, Jiang Y (2021). Synergistic effects of a combined treatment of PI3K/mTOR dual inhibitor LY3023414 and carboplatin on human endometrial carcinoma. Gynecol Oncol.

[34] Zaidi AH, Kosovec JE, Matsui D (2017). PI3K/mTOR dual inhibitor, LY3023414, demonstrates potent antitumor efficacy against esophageal adenocarcinoma in a rat model. Ann Surg.

[35] Smith MC, Mader MM, Cook JA (2016). Characterization of LY3023414, a novel PI3K/mTOR dual inhibitor eliciting transient target modulation to impede tumor growth. Mol Cancer Ther.

[36] Zhao H, Chen G, Liang H (2019). Dual PI3K/mTOR inhibitor, XL765, suppresses glioblastoma growth by inducing ER stress-dependent apoptosis. Onco Targets Ther.

[37] Rehan M (2019). Anticancer compound XL765 as PI3K/mTOR dual inhibitor: A structural insight into the inhibitory mechanism using computational approaches. PLOS ONE.

[38] Yu P, Laird AD, Du X (2014). Characterization of the activity of the PI3K/mTOR inhibitor XL765 (SAR245409) in tumor models with diverse genetic alterations affecting the PI3K pathway. Mol Cancer Ther.

[39] Zhang T, Wu DM, Luo PW (2022). CircNEIL3 mediates pyroptosis to influence lung adenocarcinoma radiotherapy by upregulating PIF1 through miR-1184 inhibition. Cell Death Dis.

[40] Fok JHL, Ramos-Montoya A, Vazquez-Chantada M (2019). AZD7648 is a potent and selective DNA-PK inhibitor that enhances radiation, chemotherapy and olaparib activity. Nat Commun.

[41] Hua H, Kong Q, Zhang H (2019). Targeting mTOR for cancer therapy. J Hematol Oncol.

[42] Simonyan L, Renault TT, Novais MJ (2016). Regulation of Bax/mitochondria interaction by AKT. FEBS Lett.

[43] Zhou B, Zhang JY, Liu XS (2018). Tom20 senses iron-activated ROS signaling to promote melanoma cell pyroptosis. Cell Res.

[44] Ghallab AM, Eissa RA, El Tayebi HM (2022). CXCR2 small-molecule antagonist combats chemoresistance and enhances immunotherapy in triple-negative breast cancer. Front Pharmacol.

[45] Guan T, Yang X, Liang H (2022). Deubiquitinating enzyme USP9X regulates metastasis and chemoresistance in triple-negative breast cancer by stabilizing Snail1. J Cell Physiol.

[46] Ghosh S (2019). Cisplatin: The first metal based anticancer drug. Bioorg Chem.

[47] Hanahan D, Weinberg RA (2011). Hallmarks of cancer: the next generation. Cell.

[48] Mansoori B, Mohammadi A, Davudian S (2017). The different mechanisms of cancer drug resistance: a brief review. Adv Pharm Bull.

[49] Campbell KJ, Tait SWG (2018). Targeting BCL-2 regulated apoptosis in cancer. Open Biol.

[50] Kale J, Kutuk O, Brito GC (2018). Phosphorylation switches Bax from promoting to inhibiting apoptosis thereby increasing drug resistance. EMBO Rep.

[51] Ganley IG, Lam du H, Wang J (2009). ULK1.ATG13.FIP200 complex mediates mTOR signaling and is essential for autophagy. J Biol Chem.

[52] Pópulo H, Lopes JM, Soares P (2012). The mTOR signalling pathway in human cancer. Int J Mol Sci.

[53] Mai FY, He P, Ye JZ (2019). Caspase-3-mediated GSDME activation contributes to cisplatin- and doxorubicin-induced secondary necrosis in mouse macrophages. Cell Prolif.

[54] Shen X, Wang H, Weng C (2021). Caspase 3/GSDME-dependent pyroptosis contributes to chemotherapy drug-induced nephrotoxicity. Cell Death Dis.

[55] Jin Z, Du X, Xu Y (2020). Structure of Mpro from SARS-CoV-2 and discovery of its inhibitors. Nature.

[56] Jiang M, Qi L, Li L (2020). The caspase-3/GSDME signal pathway as a switch between apoptosis and pyroptosis in cancer. Cell Death Discovery.

[57] Tu YC, Yeh WC, Yu HH (2022). Hedgehog suppresses paclitaxel sensitivity by regulating Akt-mediated phosphorylation of Bax in EGFR wild-type non-small cell lung cancer cells. Front Pharmacol.

[58] Wang H, Ren R, Yang Z (2021). The COL11A1/Akt/CREB signaling axis enables mitochondrial-mediated apoptotic evasion to promote chemoresistance in pancreatic cancer cells through modulating BAX/BCL-2 function. J Cancer.

[59] Yan L, Liu Y, Ma XF (2021). Triclabendazole induces pyroptosis by activating caspase-3 to cleave GSDME in breast cancer cells. Front Pharmacol.

[60] Rogers C, Erkes DA, Nardone A (2019). Gasdermin pores permeabilize mitochondria to augment caspase-3 activation during apoptosis and inflammasome activation. Nat Commun.

