# Supplementary material for: CC-115 Mediates GSDME-Dependent Pyroptosis in Lung Adenocarcinoma Through the Akt/Bax Pathway
Source: J Cancer. 2023 May 15;14(8):1350–61. doi: 10.7150/jca.83175 (PMC10240660; doi:10.7150/jca.83175)
Supplement: Supplementary file 1 — Supplementary figures. [file jcav14p1350s1.pdf]

Supplementary materials

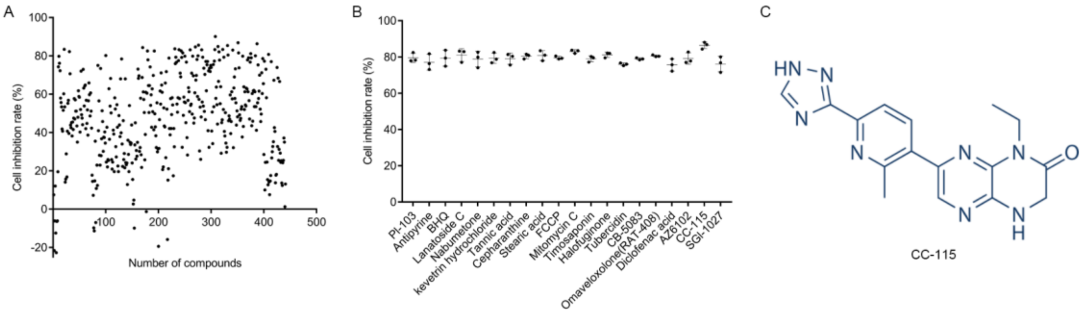

Figure S1. Screening out CC-115 based on a library of 441 pyroptosis compounds. **(A)** A549 cells were treated with 10 μM of the drug candidate library for 48 h. Each point represents the percentage of cell inhibition rate of the compounds. The cell viability was detected by CCK-8. **(B)** The top 20 compounds were confirmed in the secondary screening through CCK-8 assay. **(C)** Chemical structure of CC-115.

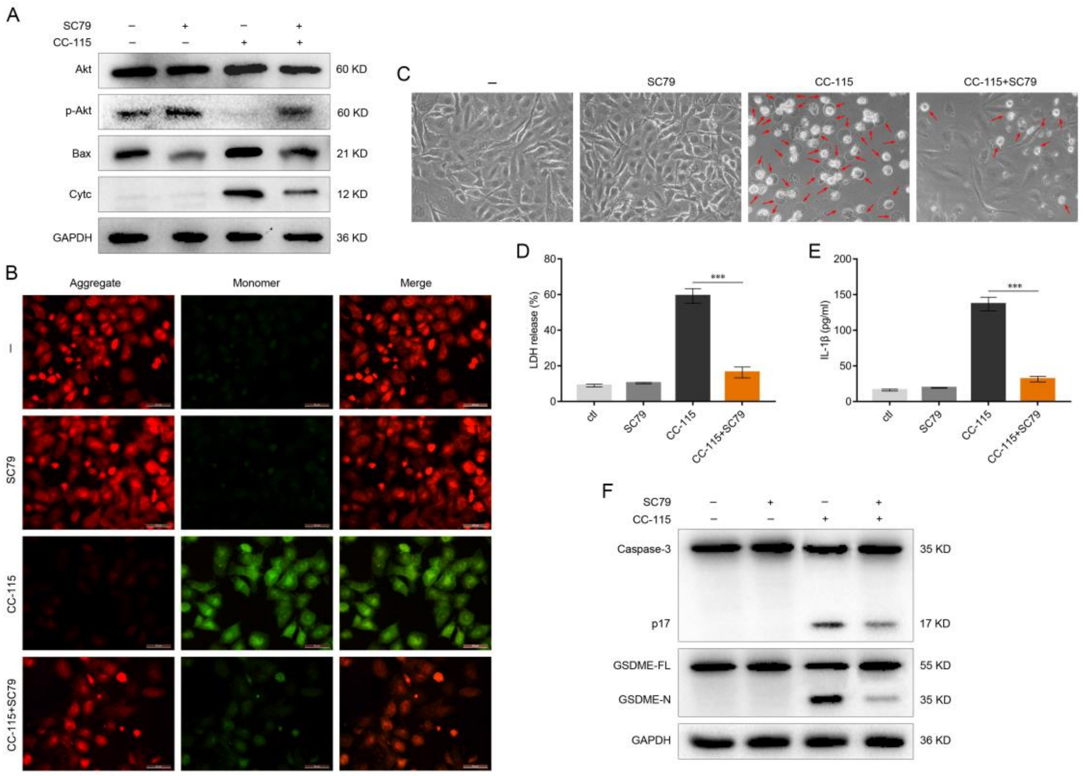

Figure S2. CC-115 promotes pyroptosis through inhibiting Akt/Bax signaling in H1650 cells. **(A)** Western blotting of Akt, Bax, and Cyt-c in H1650 cells treated with or without CC-115 (5 μM) and/or SC79 (5 μM). **(B)** Representative images of the mitochondrial membrane potential signal in H1650 cells determined using the JC-10 assay (scale bar, 50 μm). **(C)** Representative images of H1650 cells in the indicated groups (scale bar, 50 μm). **(D, E)** Release of LDH and IL-1β detected by ELISA. **(F)** Analysis of caspase-3 and GSDME expression using western blotting in the indicated groups. ****P* < 0.001, two-tailed Student's t-test.
